# Structural and molecular basis for phosphate recognition by SAR11 bacteria

**DOI:** 10.1128/mbio.01654-25

**Published:** 2025-08-13

**Authors:** Wen-Jing Zhu, Chen Wang, Li Liu, Jian-Xun Li, Hou-Qi Wang, Meng-Qi Wang, Hai-Yan Cao, Xiu-Lan Chen, Qi-Long Qin, Yu-Zhong Zhang, Mei-Ling Sun, Peng Wang

**Affiliations:** 1 MOE Key Laboratory of Evolution and Marine Biodiversity, Frontiers Science Center for Deep Ocean Multispheres and Earth System and College of Marine Life Sciences, Ocean University of China, Qingdao, China; 2 Marine Biotechnology Research Center, State Key Laboratory of Microbial Technology, Shandong University, Qingdao, China; 3 Laboratory for Marine Biology and Biotechnology, Qingdao Marine Science and Technology Center 554912 , Qingdao, China; Oregon State University, Corvallis, Oregon, USA

**Keywords:** SAR11 bacteria, phosphate transport, ABC transporter, substrate-binding protein, mechanism

## Abstract

Phosphorus is a critical limiting nutrient that constrains the survival, growth, and reproduction of marine microorganisms in nutrient-limited ecosystems. Phosphorus exists in the environment in both organic and inorganic forms, with phosphate being the predominant form of inorganic phosphorus. SAR11 bacteria, a group of oligotrophic marine bacteria, possess high-affinity transporters for limiting nutrients such as phosphate, nitrogen, and organic carbon, enabling them to dominate in nutrient-depleted environments. Despite their ecological significance, the molecular mechanisms underlying phosphate transport and metabolism in SAR11 bacteria remain elusive. Here, we investigated the phosphate transport system in *Candidatus* Pelagibacter sp. HTCC7211, a representative SAR11 bacterium, which encodes an ATP-binding cassette-type phosphate transporter, *Cp*PstSCAB. We heterologously expressed and purified *Cp*PstS, the substrate-binding component of the transporter, and determined its crystal structure in complex with phosphate to gain insights into its substrate recognition mechanism. Microscale thermophoresis binding assays demonstrated that *Cp*PstS binds phosphate with high affinity, exhibiting a dissociation constant (*K*_*d*_) of 112 nM. Phylogenetic analysis placed *Cp*PstS in a distinct branch of previously characterized PstS proteins. Structural analysis further revealed that *Cp*PstS employs a unique binding site and a distinct hydrogen-bonding network for phosphate recognition and features an expanded substrate-binding cavity, suggesting potential for organic phosphorus binding. The bioinformatic analysis further indicated that *Cp*PstS-type phosphate-binding proteins are widely distributed among SAR11 bacteria. These findings provide valuable insights into phosphorus acquisition and utilization mechanisms in SAR11 bacteria, enhancing our understanding of their adaptation to nutrient-limited marine environments.

**IMPORTANCE:**

This study provides crucial insights into phosphate acquisition in SAR11 bacteria, a key group of oligotrophic microorganisms that thrive in nutrient-limited marine ecosystems. By characterizing the unique structural features of *Cp*PstS, including its distinct hydrogen-bonding network and expanded substrate-binding cavity, this research sheds light on how SAR11 bacteria adapt to limited phosphorus availability. The discovery that *Cp*PstS may also accommodate organic phosphorus compounds broadens our understanding of microbial nutrient acquisition. These findings have significant implications for marine biogeochemical cycles and offer new perspectives on the evolution of nutrient transport mechanisms in marine microorganisms.

## INTRODUCTION

Phosphorus (P) is essential for all living organisms, contributing to the synthesis of genetic and cellular inherent components—including nucleic acids (DNA and RNA), phospholipids, and ATP—as well as cellular metabolism and energy transfer. It occurs in various natural forms, including inorganic phosphorus (Pi) and organic phosphorus (Po) compounds (1–4). Pi primarily occurs as phosphates (PO_4_^3−^) (2–4), while organic phosphorus compounds can be classified into phosphate esters, phosphonates, and organic polyphosphates, with phosphate esters being the most prevalent form (4). Among these, phosphate is the most readily assimilated form for microorganisms (5). However, phosphate is frequently a limiting nutrient in many aquatic and terrestrial ecosystems.

In prokaryotes, two main phosphate transport systems have been identified: (i) the low-affinity phosphate inorganic transport (Pit) system (6–8) and (ii) the high-affinity phosphate-specific transport (Pst) system (9, 10). The Pit system consists of a single transmembrane component that is expressed constitutively and fueled by the proton-motive force (8, 11), while the high-affinity Pst system is induced under low-phosphate conditions via the Pho regulon and functions as an ATP-binding cassette (ABC) transporter (12, 13). The high-affinity Pst system was initially characterized in *Escherichia coli* (10), *Streptomyces lividans* (14), and *Bacillus subtilis* (15, 16). This system consists of four proteins: the phosphate-specific binding protein PstS, the membrane integral proteins PstC and PstA to form a membrane channel, and an ATP-hydrolyzing protein PstB to energize phosphate transport. The Pit system is utilized in phosphate-rich environments, whereas the high-affinity Pst system, which requires more energy due to its reliance on ATP hydrolysis for phosphate transport, is employed under phosphate-depleted conditions (17). The *pstS* gene, which encodes the periplasmic phosphate-binding component of the Pst system, is nearly ubiquitous among bacterial taxa. The first crystal structure of a PstS protein was resolved from *E. coli*, providing fundamental insights into its phosphate recognition mechanism (18–20). Several phosphate-bound PstS structures have been resolved (21–26), yet no structural data exist for marine-derived PstS proteins, limiting our understanding of phosphate uptake in oligotrophic ocean environments.

**Fig 1:**
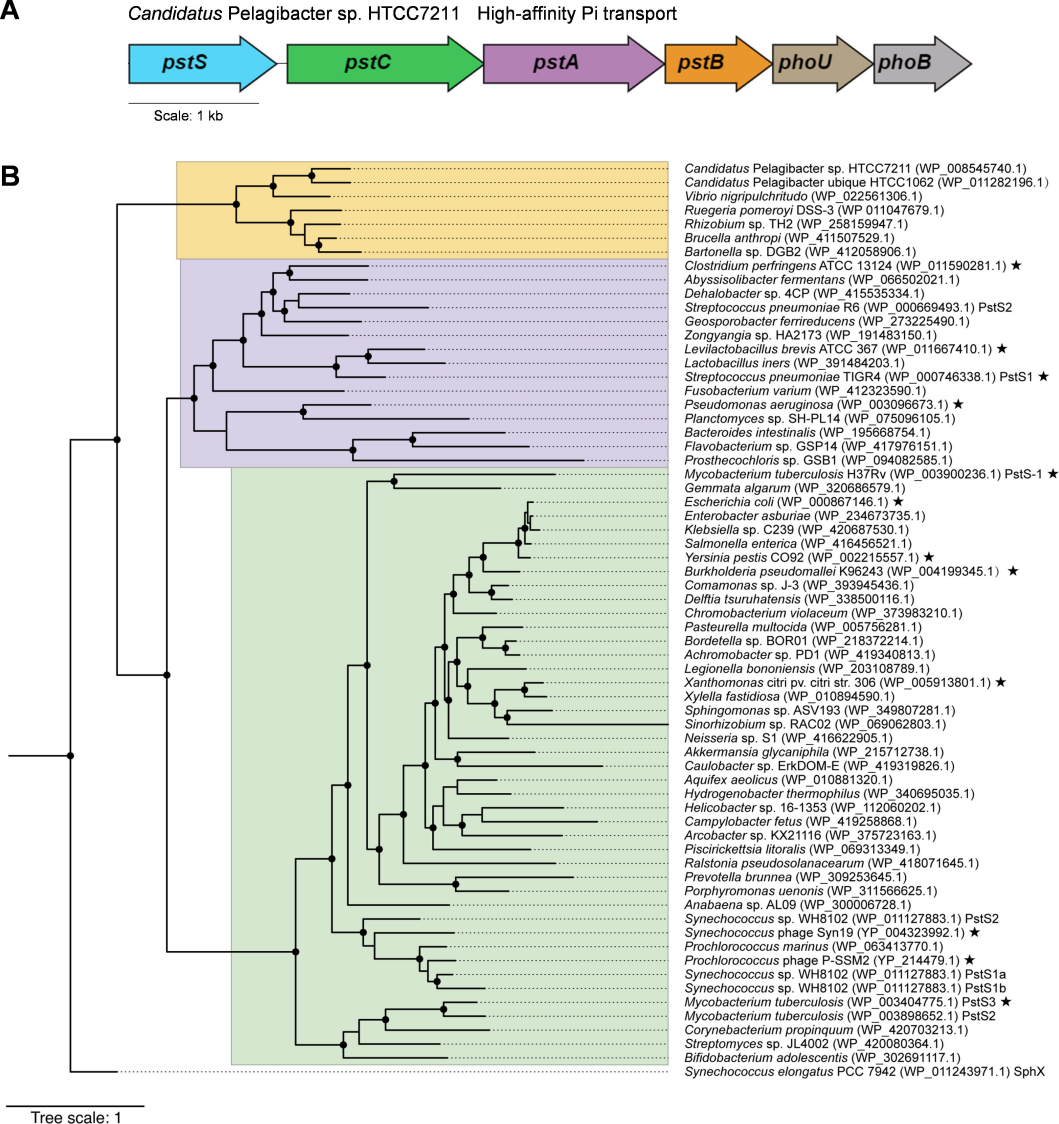
Characterization of *Cp*PstS. (**A**) High-affinity phosphate transport system of *Candidatus* Pelagibacter sp. HTCC7211. (**B**) Phylogenetic tree of different PstSs. The SphX sequence of *Synchococcus elongatus* PCC79942 was used as an outgroup. The accession number for each protein is provided in parentheses. The PstS proteins with resolved structures are labeled with black stars. Circles on branch junctions indicate bootstrap values >80%.

SAR11 bacteria, the most abundant group of heterotrophic bacteria in the ocean (27–29), are oligotrophic bacteria characterized by their small size (30), highly streamlined genome, and limited metabolic versatility (29, 31, 32). To cope with nutrient scarcity in oligotrophic environments, SAR11 bacteria rely on substrate-binding proteins (SBPs) to import specific growth substrates (33) and exhibit enhanced phosphate absorption capacity through a highly specialized phosphate transport system to overcome resource limitations (34, 35). *Candidatus* Pelagibacter sp. HTCC7211, an isolate of the SAR11 clade of marine α-proteobacteria (36), possesses a genome encoding the phosphate-specific ABC transporter (Cp*pstSCAB*), along with components of the Pho regulon (PhoB/U), which collectively regulate phosphate acquisition and homeostasis (13, 37) (Fig. 1A).

To elucidate the molecular basis of phosphate acquisition in SAR11 bacteria, we heterologously expressed and purified *Cp*PstS from *Candidatus* Pelagibacter sp. HTCC7211 in *E. coli* BL21(DE3) and determined its crystal structure in complex with phosphate. Based on structural analysis, the structural basis for the efficient recognition and binding of *Cp*PstS to phosphate is proposed. The structure reveals a previously uncharacterized binding pocket for phosphate recognition. Moreover, *Cp*PstS appears to possess potential binding capability for organophosphorus compounds. Bioinformatics analysis further indicated that *Cp*PstS-like phosphate-binding proteins are widely distributed within SAR11 bacteria. Together, these findings provide new insights into phosphorus uptake strategies in oligotrophic marine bacteria and highlight structural adaptations enabling nutrient scavenging in phosphorus-depleted environments.

## RESULTS AND DISCUSSION

### Sequence analysis of *Cp*PstS

The Cp*pstS* gene of *Candidatus* Pelagibacter sp. HTCC7211 is 1,035 bp in length, encoding a 344-amino acid residue phosphate-binding protein (*Cp*PstS; GenBank accession no. WP_008545740.1), and is the only *pstS* gene copy present in the genome. Conservation domain analysis indicated that *Cp*PstS belongs to the type 2 periplasmic binding fold superfamily, which includes SBPs involved in high-affinity transport of soluble nutrients via ABC transporters. These proteins function in the uptake of various soluble substrates such as phosphate, sulfate, amino acids, and sugars (38). Among PstS proteins with resolved structures, the closest homolog to *Cp*PstS is PstS from *Clostridium perfringens* ATCC 13124, with a protein sequence identity of 26%.

PstS, the substrate-binding component of the high-affinity phosphate ABC transporter, is widely distributed. A search of the National Center for Biotechnology Information (NCBI) protein database showed that PstSs are mainly found in the classes Gammaproteobacteria, Alphaproteobacteria, and Betaproteobacteria of the phylum Proteobacteria, as well as in the class Actinobacteria of the phylum Actinobacteriota, and are also widely distributed in the phyla Firmicutes, Bacteroidota, and Cyanobacteriota. We selected PstS sequences derived from representative species of different classes for the construction of the phylogenetic tree. In addition, previous studies have characterized the structure and function of PstS proteins from *Prochlorococcus* phage P-SSM2 and *Synechococcus* phage Syn19 (26). Since phage-derived PstS may have important relationships with bacterial PstS, we selected 65 PstS sequences derived from representative species of different classes, along with PstSs from *Prochlorococcus* phage P-SSM2 and *Synechococcus* phage Syn19, including both resolved and unresolved structures, to construct the phylogenetic tree (18, 21–25) (Fig. 1B). In several bacterial genomes, multiple paralogous copies of the *pstS* gene were observed. Notably, *Mycobacterium tuberculosis* and *Synechococcus* sp. WH8102 possesses three distinct *pstS* homologs (23, 39–41), whereas *Streptococcus pneumoniae* contains two (41). These homologs have been specifically labeled in the phylogenetic tree (Fig. 1B). The phylogenetic tree revealed that PstSs are grouped into several distinct clades, suggesting that they belong to different subfamilies (Fig. 1B). Notably, the clade represented by *Cp*PstS forms a separate cluster, and the structure and phosphate recognition mechanisms of this group of PstSs remain unexplored.

### Purification and activity assay of *Cp*PstS

*Cp*PstS contains a 23-residue signal peptide predicted by the SignalP 5.0 server. *Cp*PstS without the signal peptide was expressed in *E. coli* BL21(DE3). The purified recombinant *Cp*PstS is approximately 35 kDa (Fig. S1A), consistent with its predicted molecular mass of ~35 kDa. Gel filtration analysis showed that the molecular weight of *Cp*PstS in solution is larger than 29 kDa and smaller than 44 kDa, indicating that *Cp*PstS presents as a monomer in solution (Fig. S1B).

**Fig 2:**
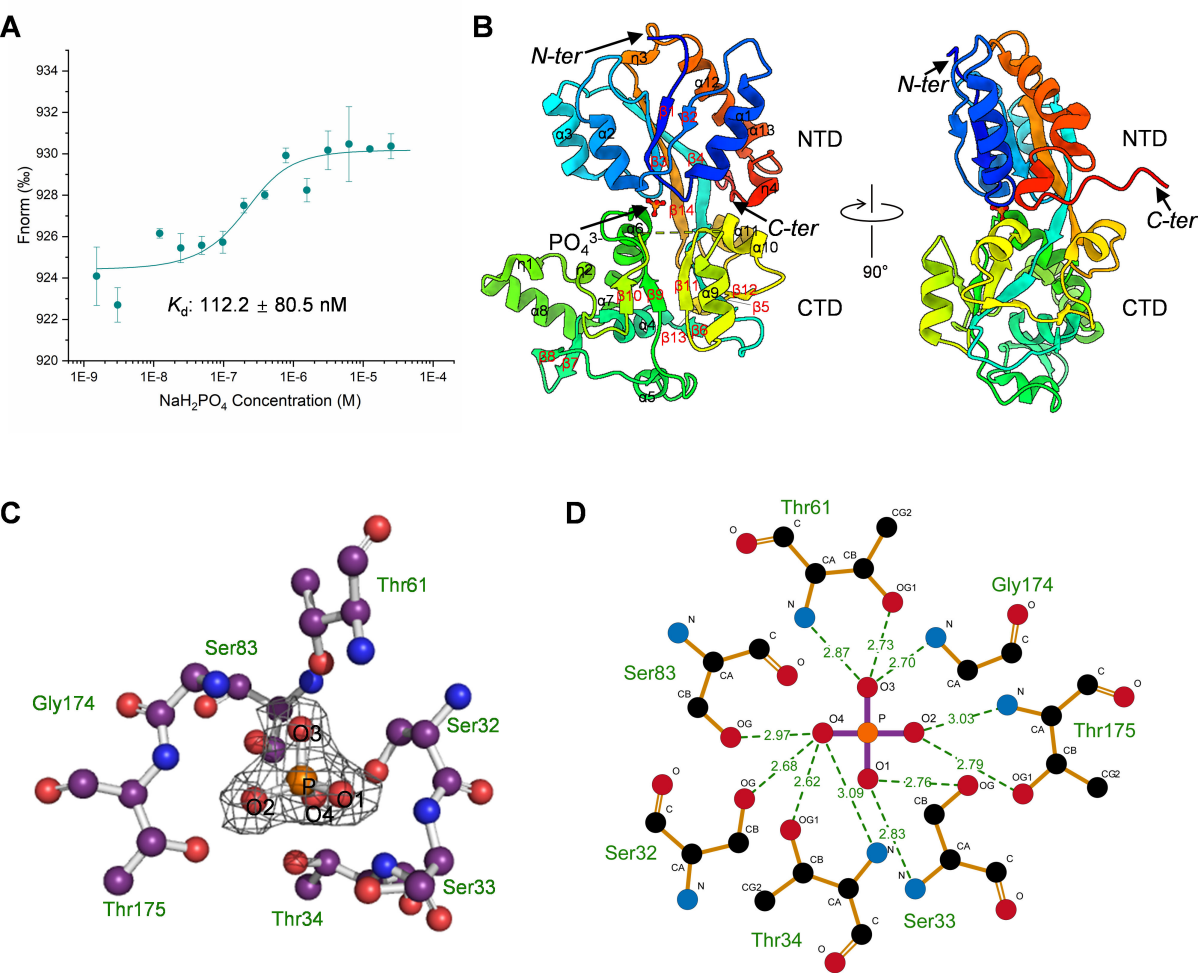
Phosphate binding site of *Cp*PstS. (**A**) MST analysis of phosphate binding to *Cp*PstS. The NaH_2_PO_4_ is titrated from 1.53 nM to 50 µM. (**B**) The overall structure of *Cp*PstS, with the phosphate molecule shown as an orange ball-and-stick model. (**C**) Stereo view of the electron density of the phosphate bound in *Cp*PstS. (**D**) Hydrogen bonds between the phosphate molecule and *Cp*PstS, with hydrogen bonds shown by green dashes.

To analyze the binding affinity of *Cp*PstS to phosphate, the microscale thermophoresis (MST)-binding assay was performed using the Monolith NT.115 instrument (NanoTemper Technologies GmbH). Based on previous studies showing that SAR11_1179 has a high binding affinity to phosphate (33), we purified the *Cp*PstS protein under denaturing conditions and refolded it for the MST assay. The NaH_2_PO_4_ is titrated from 1.53 nM to 50 µM. The change in the thermophoretic signal leads to a *K*_*d*_ of 112.2 ± 80.5 nM (Fig. 2A). The high-affinity phosphate-binding affinity of *Cp*PstS is consistent with that previously reported for SAR11_1179 (33). To further investigate the molecular mechanism underlying the high-affinity phosphate binding of *Cp*PstS, we subsequently conducted structural biology studies on *Cp*PstS.

### Overall structure of *Cp*PstS

We purified *Cp*PstS and visualized the structure of P12_1_1 space group at 2.0 Å resolution (Table 1; Protein Data Bank [PDB] code: 9JWY). Although no additional phosphate was added during the protein purification, crystallization, and data collection process, clear phosphate densities were observed in the resolved structure (Fig. 2B and C). Given *Cp*PstS’s high-efficiency potential in recognizing and binding phosphate, we hypothesize that phosphate ions were already specifically bound within the binding pocket during protein expression and purification. Furthermore, this result suggests that *Cp*PstS possesses an exceptionally high binding affinity for phosphate, enabling it to recognize and bind phosphate even at extremely low environmental concentrations.

**TABLE 1:** Diffraction data and refinement statistics of *Cp*PstS/phosphate complex

Parameter(s)	Value(s)^*a*^
Diffraction data	
Space group	P12_1_1
Unit cell	
*a*, *b*, and *c* (Å)	38.2698, 46.8699, and 82.99
α, β, and γ (°)	90, 102.99, and 90
Resolution range (Å)	40.43–2.0 (2.071–2.0)
Completeness (%)	95.17 (91.76)
*R*_merge_^*b*^	0.095
*I*/σ*I*	10.5 (5.4)
CC_1/2_	0.990 (0.976)
Refinement statistics	
*R* factor	0.1770
Free *R* factor	0.2172
Wilson *B*-factor	16.07
Root-mean-square deviation from ideal geometry	
Bond length (Å)	0.011
Bond angle (°)	1.43
Ramachandran plot (%)	
Favored	97.98
Allowed	2.02
Overall *B* factor (Å^2^)	18.23

^*a*^ Numbers in parentheses refer to data in the highest-resolution shell.

^*b*^ *R*_merge_ = ∑_hkl_ ∑*_i_*|*I*(hkl)*_i_* – <*I*(hkl)>|/∑hkl ∑*_i_*<*I*(hkl)*_i_*>.

The structure of *Cp*PstS/phosphate complex shows that each asymmetric unit contains one *Cp*PstS molecule, and each *Cp*PstS molecule binds with one phosphate molecule (Fig. 2B). *Cp*PstS possesses the typical structural characteristics of type 2 periplasmic binding fold superfamily proteins. It consists of two domains, an N-terminal domain (NTD) and a C-terminal domain (CTD), linked by a hinge region. Due to the poor density at residues 224–227, these three residues were not built during the structure determination. The NTD (residues 24–111 and 279–324) consists of five parallel β-strands (β1–β4 and β14), which form a highly twisted β-sheet flanked on both faces by five α-helices (α1–α3, α12, and α13). The CTD (residues 112–224 and 228–278) has a similar topology to the NTD, with a central core of five parallel β-strands (β1 and β9–β12) flanked by eight α-helices (α4–α11) and two pairs of parallel β-strands (β6 and β13, β7 and β8) at the edge. The hinge region (residues 102–111 and 112–117) is composed of two β-strands (β4 and β5) originating from the two spherical domains (NTD and CTD), linking them together and forming a Venus flytrap-like topology. The phosphate molecule is bound in the cleft between the two domains (Fig. 2B).

Structural comparisons between *Cp*PstS and previously determined PstS proteins from various bacterial species revealed root-mean-square deviation (RMSD) values ranging from 1.0 to 1.3 Å, indicating a high degree of structural similarity. The closest match was observed with *Streptococcus pneumoniae* TIGR4 PstS1 (PDB ID: 4LAT), with an RMSD of 1.067 Å, whereas the greatest deviation was found in comparison with *Xanthomonas citri* pv. citri str. 306 PstS, with an RMSD of 1.271 Å. The RMSD between *Cp*PstS and the first structurally characterized PstS from *E. coli* (also PDB ID: 1IXH) is 1.217 Å (18). These findings indicate that *Cp*PstS adopts a conserved overall fold typical of PstS proteins, while also exhibiting subtle conformational variations that may reflect species-specific structural adaptations.

### Key residues of *Cp*PstS for phosphate recognition and binding

Structural analysis revealed that seven residues are involved in the recognition and binding of phosphate: Ser32, Ser33, Thr34, Thr61, Ser83, Gly174, and Thr175 (Fig. 2C). These residues collectively form 11 hydrogen bonds with the phosphate molecule, anchoring it securely in the cleft between the NTD and the CTD of *Cp*PstS (Fig. 2B and D). Among these, five hydrogen bonds are formed with backbone N-H groups (Ser33, Thr34, Thr61, Gly174, and Thr175), while six are formed with side chain O-H groups (Ser32, Ser33, Thr34, Thr61, Ser83, and Thr175; Fig. 2D and Table S1). The hydrogen bond distances range from 2.62 Å to 3.03 Å, with the shortest bond being formed between the phosphate O4 atom and the Thr34 OG1 group (Table S1).

Multiple sequence alignment analysis shows that the seven key residues involved in phosphate binding are highly conserved among the PstS sequences from SAR11 bacteria (Fig. S2). Furthermore, analysis of PstS sequences from non-SAR11 bacteria within the same phylogenetic branch revealed that the amino acids involved in phosphate binding are also relatively conserved (Fig. S3). These findings indicate that the key residues responsible for phosphate binding are completely conserved within SAR11 bacteria and relatively conserved among bacteria on the same phylogenetic branch as *Cp*PstS. This suggests that these PstS proteins likely employ a similar mechanism for recognizing and binding phosphate.

**Fig 3:**
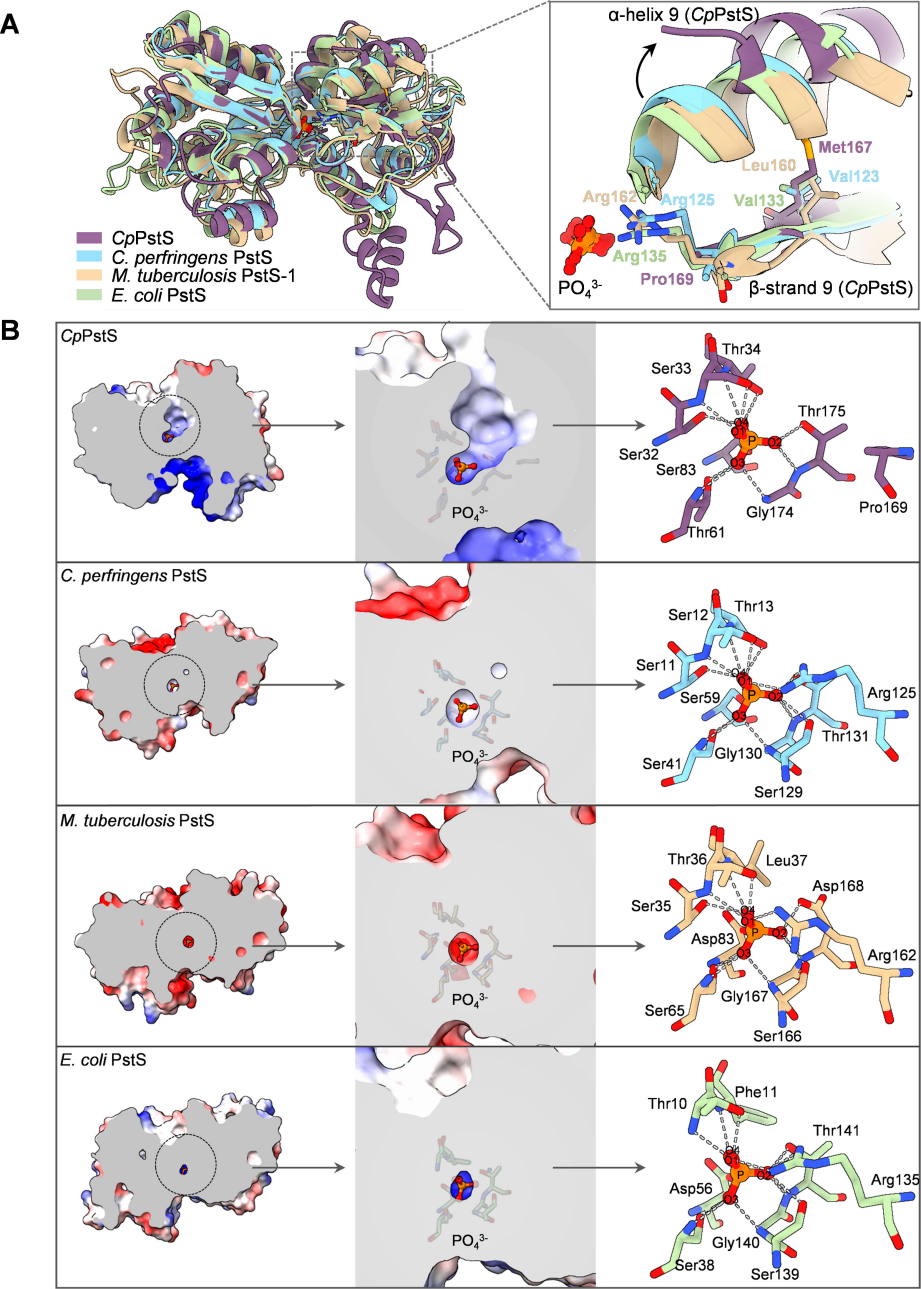
Structural comparison of *Cp*PstS with PstS homologs from *C. perfringens* ATCC 13124, *M. tuberculosis* H37Rv, and *E. coli*. (**A**) Structural alignment of the four structures. The structures of *Cp*PstS, *C. perfringens* PstS (PDB code: 4Q8R), *M. tuberculosis* PstS-1 (PDB code: 1PC3), and *E. coli* PstS (PDB code: 1IXH) are represented as cartoons and colored in purple, blue, beige, and green, respectively. (**B**) Electrostatic surface and substrate-binding cavity of the four structures. Residues involved in phosphate binding are shown as sticks, and phosphate molecules are depicted as orange ball-and-stick models.

Compared to phosphate-binding proteins from different phylogenetic branches, PstSs represented by *Cp*PstS exhibit notable differences in phosphate recognition and binding. The most significant distinction lies in the absence of a critical hydrogen bond interaction contributed by an arginine residue (Table S1). In the corresponding positions of Arg125 in *C. perfringens* ATCC 13124 PstS (PDB code: 4Q8R) (25) and Arg162 in *M. tuberculosis* H37Rv PstS-1 (PDB code: 1PC3) (24), which represent the other two branches, Arg is replaced by Pro169 in *Cp*PstS (Fig. 3). Pro169 is a residue incapable of forming hydrogen bond interactions with phosphate. Since the first PstS structure was resolved in *E. coli* (18–20), we also compared the key residues involved in phosphate binding between the *E. coli* PstS and *Cp*PstS. In *E. coli* PstS, an arginine exists in the corresponding site of Pro169 in *Cp*PstS (Fig. 3 and Table S1), suggesting that *Cp*PstS may have evolved a distinct hydrogen-bonding network for phosphate binding. The multiple sequence alignment results show that these key residues are relatively conserved within their respective branches (Fig. S3 to S5).

It has been reported that a single amino acid substitution, where threonine replaces serine in the binding pocket of *Synechococcus* sp. WH8102 PstS1b, compared to PstS1a and PstS2, is likely responsible for its higher phosphate-binding affinity (41). The phosphate-binding affinity of *Cp*PstS was substantially higher than that of *Synechococcus* sp. WH8102 PstS, which have been reported to exhibit *K*_*d*_ values ranging from 0.4 to 4.3 µM (PstS1b, *K*_*d*_ = 0.44 µM; PstS1a, *K*_*d*_ = 3.3 µM; PstS2, *K*_*d*_ = 4.3 µM). To determine whether *Cp*PstS contains a similar substitution observed in PstS1b, we performed multiple sequence alignment (Fig. S6). The results revealed that the corresponding residue in *Cp*PstS is a serine, suggesting that the high phosphate-binding affinity of *Cp*PstS may not be due to the same mechanism proposed in *Synechococcus*. Instead, other structural features such as the overall hydrogen-bonding network and the geometry of the binding pocket may play a more significant role in the high-affinity phosphate binding of *Cp*PstS. This also suggests that PstS proteins from different branches in the phylogenetic tree may have differences in their phosphate-binding mechanisms.

### The expanded substrate-binding cavity of *Cp*PstS

Apart from the differences in hydrogen bond interactions for phosphate recognition and binding, the binding cavity of *Cp*PstS is also notably distinct. We systematically searched the PDB for all available structures of PstS proteins in complex with phosphate and conducted a comparative analysis of their substrate-binding cavity with that of *Cp*PstS to gain insights into potential structural differences. It was observed that *Cp*PstS has a significantly larger substrate-binding cavity compared to the structures of *C. perfringens* PstS, *M. tuberculosis* PstS-1, *E. coli* PstS, and other PstS from different bacteria (Fig. 3B and Fig. S7). In addition to bacterial PstS proteins, we also included the structures of *Prochlorococcus* phage P-SSM2 and *Synechococcus* phage Syn19 for comparison of the substrate-binding cavity (26). The results show that they also possess relatively small substrate-binding cavities (Fig. S7). Structural comparison reveals that α-helix 9 (residues 229–236) near the substrate-binding cavity in *Cp*PstS rotated approximately 20° relative to the corresponding α-helix in the *C. perfringens* PstS, *M. tuberculosis* PstS-1, and *E. coli* PstS structures (Fig. 3A). This rotation substantially enlarges the substrate-binding cavity. The rotation of α-helix 9 may be associated with Met167 on β-strand 9. Compared to the smaller side chains of Val123 in *C. perfringens* PstS, Leu160 in *M. tuberculosis* PstS-1, and Val133 in *E. coli* PstS, the larger side chain of Met167 likely influences the conformation of α-helix 9. Additionally, the previously mentioned substitution of Arg with Pro in *Cp*PstS (Pro169), which reduces the number of substrate-binding hydrogen bonds, also contributes to the enlargement of the substrate-binding cavity. Proline’s side chain is significantly smaller than that of arginine, further expanding the cavity.

### The binding activity of *Cp*PstS for organic phosphorus compounds

**Fig 4:**
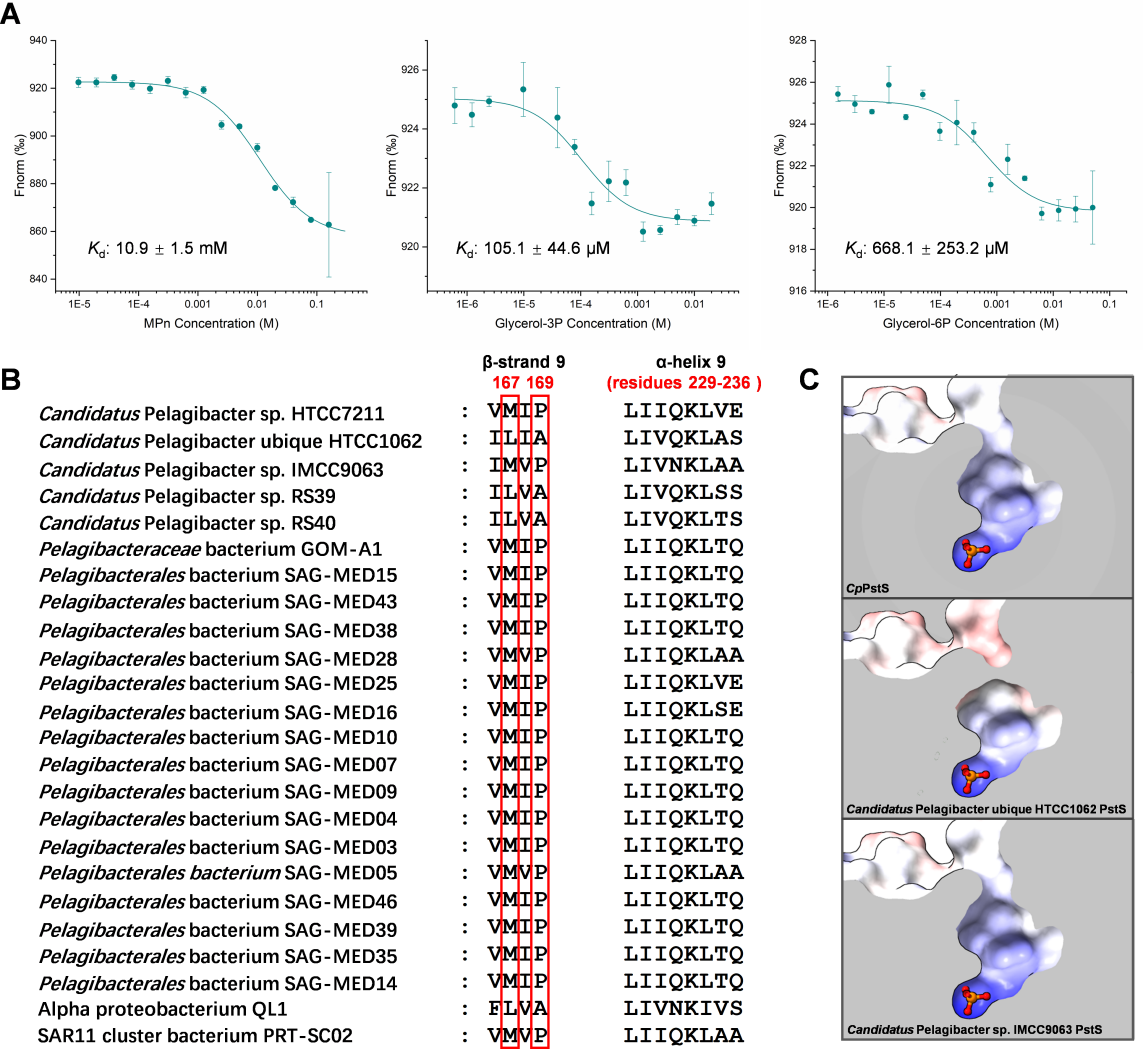
Binding of *Cp*PstS to organic phosphorus compounds. (**A**) MST analysis of *Cp*PstS binding to methyl phosphonate (Mpn), glycerol-3-phosphate (Glycerol-3P), and glucose-6-phosphate (Glucose-6P). (**B**) Multiple sequence alignment of SAR11 PstSs, highlighting key residues influencing cavity dimensions. The residues in the red box are the key residues that possibly affect the dimensions of the substrate-binding cavity. (**C**) Predicted phosphate-binding cavities of *Candidatus* Pelagibacter ubique HTCC1062 and *Candidatus* Pelagibacter sp. IMCC9063 PstSs.

Most phosphorus transporters exhibit high substrate specificity (17). Previous studies have shown that PstSCAB specifically transports inorganic phosphate (10, 41, 42). However, a few exceptions suggest a broader substrate range in some systems. Notably, the phosphonate transport system PhnCDE has been reported to mediate not only phosphonate uptake but also inorganic phosphate transport under certain conditions (43, 44). These findings suggest that some phosphorus transporters may have relaxed substrate specificity and accommodate structurally diverse compounds. We therefore speculate that the larger substrate-binding cavity of *Cp*PstS may allow it to bind to both phosphate and larger organic phosphorus compounds. To test this hypothesis, we conducted an MST-binding assay. MST analysis revealed that the *K*_*d*_ values of *Cp*PstS for glycerol-3-phosphate (Glycerol-3P), glucose-6-phosphate (Glucose-6P), and methyl phosphonate (Mpn) are 105.1 ± 44.6 µM, 668.1 ± 253.2 µM, and 10.9 ± 1.5 mM, respectively (Fig. 4A). The result revealed that *Cp*PstS exhibits binding activity toward different organic phosphorus compounds, although the *K*_*d*_ values are relatively high. We speculate that the compounds we selected may not represent the optimal organic phosphorus substrate for *Cp*PstS. Considering the size of the binding pocket, it is possible that *Cp*PstS may exhibit higher affinity for other organic phosphorus compounds, such as nucleotides, phosphoserine, or inositol phosphates. Nevertheless, these results provide stronger support for our speculation that *Cp*PstS has the potential to accommodate and bind phosphorus substrates. Based on these experimental results, we suggest that the substrate-binding cavity of *Cp*PstS might possess the versatility to bind not only inorganic phosphate but also various organic phosphorus compounds, particularly under conditions where inorganic phosphate is scarce. In fact, organic phosphorus has been shown to dominate the marine phosphorus pool in many oligotrophic regions (1, 45–47), further highlighting the potential significance of *Cp*PstS’s broad substrate-binding capacity.

Multiple sequence alignment revealed that the key residues Met167 and Pro169, which contribute to the expanded substrate-binding cavity of *Cp*PstS, are relatively conserved within the SAR11 bacteria (Fig. 4B). Furthermore, at the structural level, modeled structures of PstSs from two other representative SAR11 bacteria, *Candidatus* Pelagibacter sp. HTCC1062 and *Candidatus* Pelagibacter sp. IMCC9063, based on the *Cp*PstS template, also exhibit large substrate-binding cavities similar to that of *Cp*PstS (Fig. 4C). These findings suggest that PstSs in SAR11 bacteria likely share a larger binding cavity and may possess the ability to bind organic phosphorus compounds.

### Conclusion

Phosphorus is an essential nutrient that plays a fundamental role in the survival, growth, and reproduction of marine microorganisms (48, 49). Phosphate is the most important form of inorganic phosphorus. In this study, we elucidated the structural basis by which SAR11 bacteria—the most abundant and widespread bacterial group in the ocean—recognize and bind phosphate. Phylogenetic analysis revealed that PstS proteins from the SAR11 bacteria form a distinct evolutionary branch and utilize a unique set of amino acids and hydrogen bonds to bind phosphate. Furthermore, we discovered that SAR11-derived PstS proteins possess a larger phosphate-binding cavity, which may enable them to bind not only phosphate but also other larger phosphorus compounds. This implies that their Pst system has the potential to transport phosphorus resources beyond inorganic phosphate. However, further investigations are required at the membrane transport level. In summary, this study provides new insights into how SAR11 bacteria absorb and utilize phosphorus, shedding light on their adaptation to nutrient-limited marine environments.

## MATERIALS AND METHODS

### Bacterial strains and growth conditions

Strains and plasmids used in this study are listed in Table S2. *E. coli* DH5α was grown in lysogeny broth (LB) medium at 37°C. *E. coli* BL21(DE3) was cultured in LB medium or M9 minimal medium at 37°C.

### Gene cloning, protein expression, and protein purification

Using primers Cp*pstS*-F/Cp*pstS*-R, the Cp*pstS* gene was PCR amplified from the *Candidatus* Pelagibacter sp. HTCC7211 strain and inserted into the *NdeI*/*XhoI* restriction sites of the pET-22b vector containing a C-terminal His-tag (Table S3). The *Cp*PstS and the selenomethionine (SeMet) derivative of *Cp*PstS were expressed in *E. coli* BL21(DE3) cells under different culture conditions. For *Cp*PstS protein expression for MST-binding assay, cells were cultured at 37°C in LB medium supplemented with 100 µg/mL ampicillin to an OD_600_ of 0.8–1.0, and induction was initiated with 0.35 mM isopropyl-β-D-thiogalactopyranoside (IPTG) at 15°C for 16 hours. For SeMet-*Cp*PstS protein expression for crystallization, the SeMet-*Cp*PstS protein was overexpressed in *E. coli* BL21(DE3) using M9 minimal medium supplemented with selenomethionine, lysine, leucine, valine, isoleucine, threonine, and phenylalanine (50). The overexpression was induced with 0.35 mM IPTG at 15°C for 16 hours. The purification of *Cp*PstS for MST-binding assay was under denaturing conditions, cultured *E. coli* BL21(DE3) cells were collected by centrifugation, resuspended in lysis buffer (40 mM Tris-HCl [pH 8.0], 200 mM NaCl, 5% [vol/vol] glycerol, 2 mM EDTA, and 0.1 mM phenylmethanesulfonyl fluoride), and fractured by pressure crusher (JNBIO, China). The *Cp*PstS protein was first purified by affinity chromatography on a Ni^2+^-NTA column (Qiagen, Germany), washed with denaturing Ni binding buffer (8 M urea, 20 mM Tris, 250 mM NaCl, and 20 mM imidazole, pH 8.0) and eluted with denaturing Ni elution buffer (8 M urea, 20 mM Tris, 250 mM NaCl, and 250 mM imidazole, pH 8.0). The denatured *Cp*PstS protein was transferred to 10 kDa molecular weight cutoff (MWCO) SnakeSkin dialysis tubing (Thermo Scientific) and dialyzed at 4°C for 24 hours, during which the dialysis solution was gradually replaced with decreasing concentrations of urea, and finally with urea-free dialysis buffer (20 mM Tris and 150 mM NaCl, pH 8.0). The *Cp*PstS protein was collected and exchanged into the buffer containing 40 mM MES (pH 7.2) for the MST-binding assay. The purification of SeMet-*Cp*PstS for crystallization was under native conditions, and cultured *E. coli* BL21(DE3) cells were collected, resuspended, and lysed in lysis buffer by pressure crusher (JNBIO, China). The SeMet-*Cp*PstS protein was first purified by affinity chromatography on a Ni^2+^-NTA column (Qiagen, Germany), washed with 30 mM imidazole, 40 mM Tris-HCl (pH 8.0), 200 mM NaCl, and 5% (vol/vol) glycerol, and then eluted with buffer containing 250 mM imidazole, 40 mM Tris-HCl (pH 8.0), 200 mM NaCl, and 5% (vol/vol) glycerol. Then the purified proteins were fractionated by gel filtration on a Superdex-200 column (GE Healthcare, America) in a buffer containing 20 mM Tris-HCl (pH 8.0) and 200 mM NaCl.

### Crystallization and data collection

The purified *Cp*PstS proteins were concentrated to 7 mg/mL in 10 mM Tris-HCl (pH 8.0) and 100 mM NaCl. Initial crystallization trials for *Cp*PstS were conducted using the sitting-drop vapor diffusion method at 18°C. The reservoir solution was mixed with the *Cp*PstS solution in a ratio of 1:1. High-quality diffraction crystals of *Cp*PstS were obtained in hanging drops containing 0.2 M ammonium chloride, 0.1 M Tris (pH 8.0), and 24% (wt/vol) polyethylene glycol 6,000 at 18°C after 2 weeks. X-ray diffraction data were collected on the BL18U1 and BL19U1 beamlines at the Shanghai Synchrotron Radiation Facility. The initial diffraction data sets were processed using the HKL3000 program (51).

### Structure determination and refinement

The crystals of the SeMet-*Cp*PstS/phosphate complex belong to the P12_1_1 space group. The structure was determined using single-wavelength anomalous dispersion phasing with a selenomethionine derivative, processed with Phenix 1.19.2-4158 (52). Refinement of the structure was performed using WinCoot and Phenix 1.19.2-4158 (52, 53). The quality of the final model is summarized in Table 1. All the structure figures were made using the LigPlot^+^ (54), the PyMOL program (http://www.pymol.org/), and the ChimeraX program (https://www.cgl.ucsf.edu/chimerax/).

### MST-binding assay

MST technology was used to quantify the binding affinities of NaH₂PO₄, Mpn, Glycerol-3P, and Glucose-6P to *Cp*PstS. Purified *Cp*PstS was labeled with the Large Volume Protein Labeling Kit RED-Tris-NTA 2nd Generation (NanoTemper Technologies GmbH). Compounds were diluted in a twofold dilution gradient concentration and mixed with the labeled *Cp*PstS at 25°C in buffer containing 40 mM MES (pH 7.2) and 0.005% Tween-20. The mixed samples were then loaded into Monolith NT.115 Series capillaries (NanoTemper Technologies GmbH) and analyzed using a Monolith NT.115 instrument (NanoTemper Technologies GmbH). The *K*_*d*_ values were calculated by fitting the MST data using the MO.Affinity Analysis (x86) software, and the plots were created in Origin 2018 software.

### Bioinformatics analysis

SignalP 5.0 was used to predict the signal peptide of *Cp*PstS. The molecular mass of *Cp*PstS was calculated by ExPASy (https://web.expasy.org/compute_pi/). The NCBI Conserved Domain Database was used to analyze the conserved domain structure of *Cp*PstS. The phosphate-binding proteins were searched in the GenBank Database. The organisms and GenBank accession numbers of phosphate-binding proteins from SAR11 bacteria are listed in Table S4. PstS sequences were aligned using the L-INS-I option of MAFFT (55), and the phylogenetic tree was inferred using IQ-Tree (56). The final phylogenetic tree was visualized using FigTree v1.4.4 (http://tree.bio.ed.ac.uk/software/figtree/). Multiple sequence alignment was visualized by ESPript 3.0 (57).

## Data Availability

The atomic coordinates and structure factors of *Cp*PstS have been deposited in the Protein Data Bank (PDB) under accession code 9JWY.

## SUPPLEMENTAL MATERIAL

Supplemental figures and tables mbio.01654-25-s0001.docx Fig. S1 to S7; Tables S1 to S4.

ASM does not own the copyrights to Supplemental Material that may be linked to, or accessed through, an article. The authors have granted ASM a non-exclusive, world-wide license to publish the Supplemental Material files. Please contact the corresponding author directly for reuse.
