# Supplementary material for: Structural and molecular basis for phosphate recognition by SAR11 bacteria
Source: mBio. 2025 Aug 13;16(9):e01654-25. doi: 10.1128/mbio.01654-25 (PMC12421899; doi:10.1128/mbio.01654-25)
Supplement: Supplemental figures and tables — Fig. S1 to S7; Tables S1 to S4. [file mbio.01654-25-s0001.docx]

**SUPPLEMENTAL MATERIAL**

**
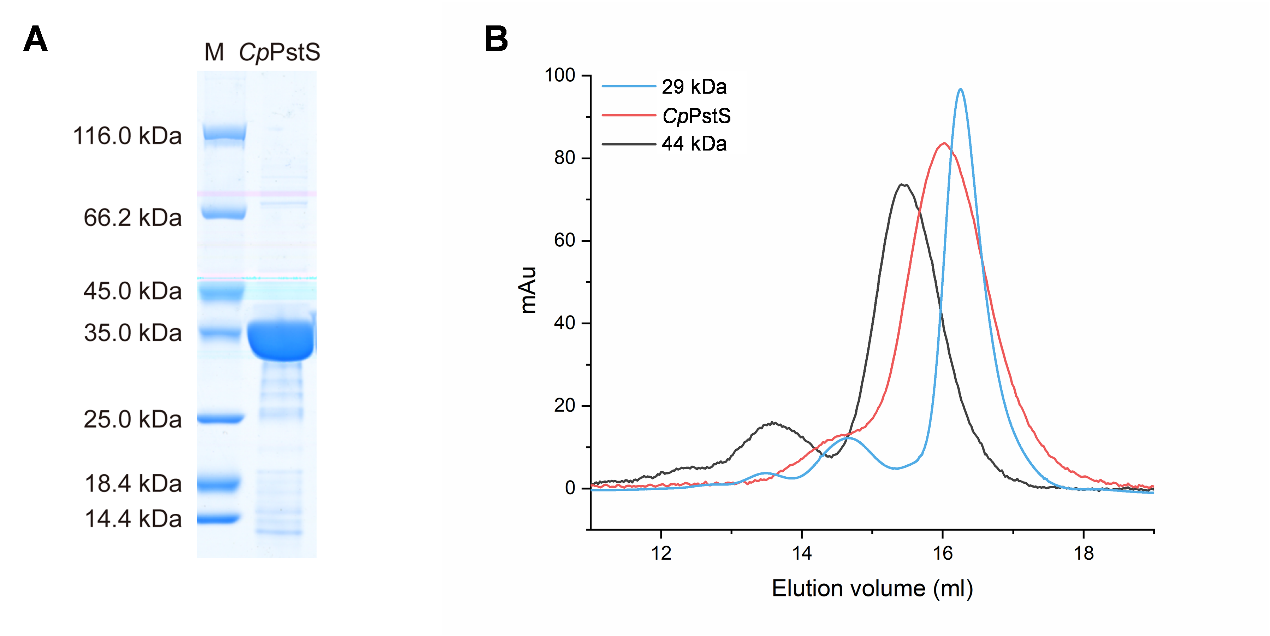
**

**Fig. S1 Characterization of purified *Cp*PstS. (A)** SDS-PAGE analysis of the purified recombinant *Cp*PstS. **(B)** Gel filtration analysis of the purified recombinant *Cp*PstS. Ovalbumin (44 kDa; GE Healthcare) and Carbonic anhydrase (29 kDa; GE Healthcare) were used as protein size markers.

**
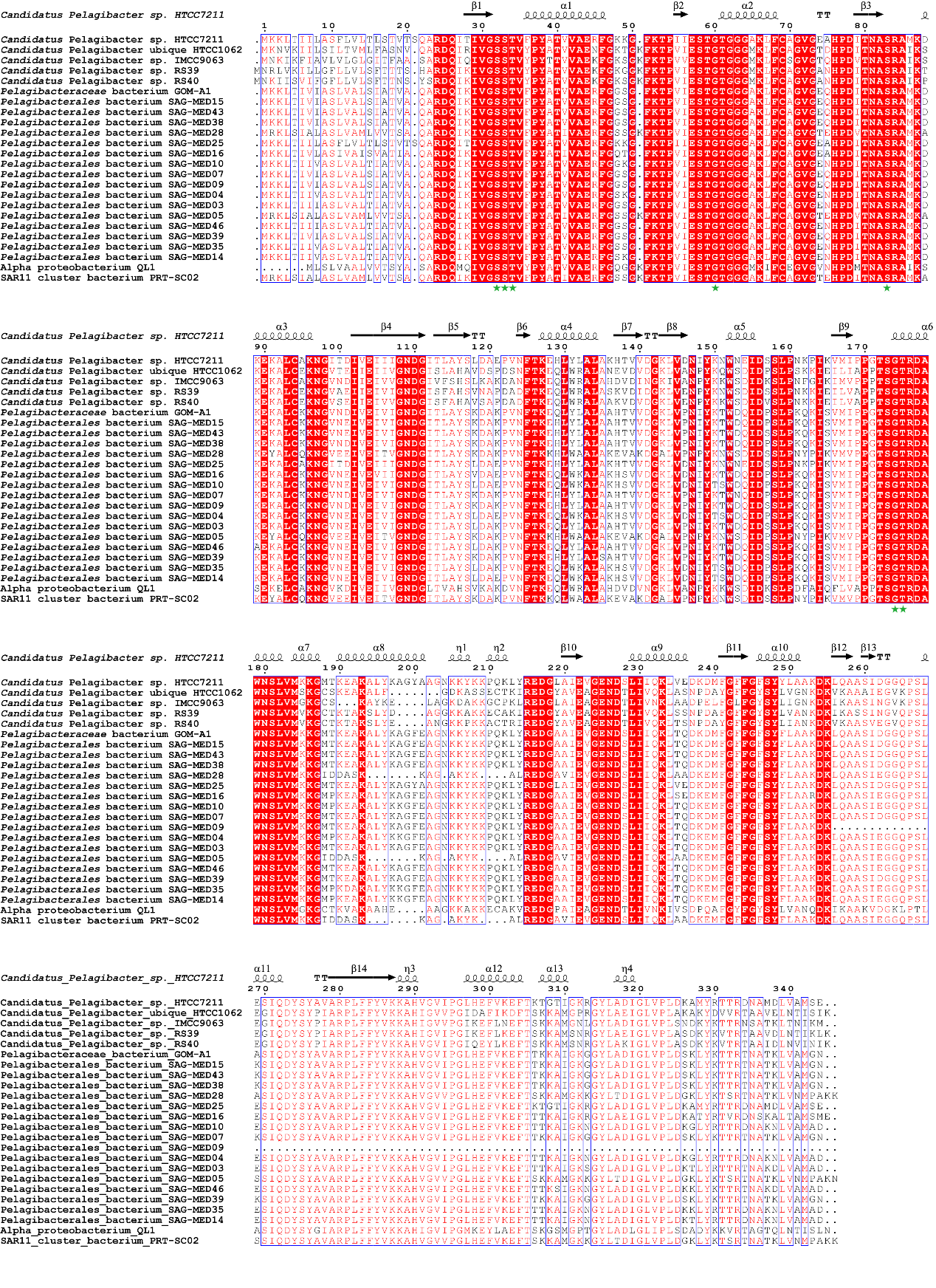
**

**Fig. S2 Multiple sequence alignment of PstSs from SAR11 bacteria.** The secondary structural elements of *Cp*PstS are shown at the top. The key residues involved in phosphate binding of *Cp*PstS are marked with green stars at the bottom of the sequence alignment.


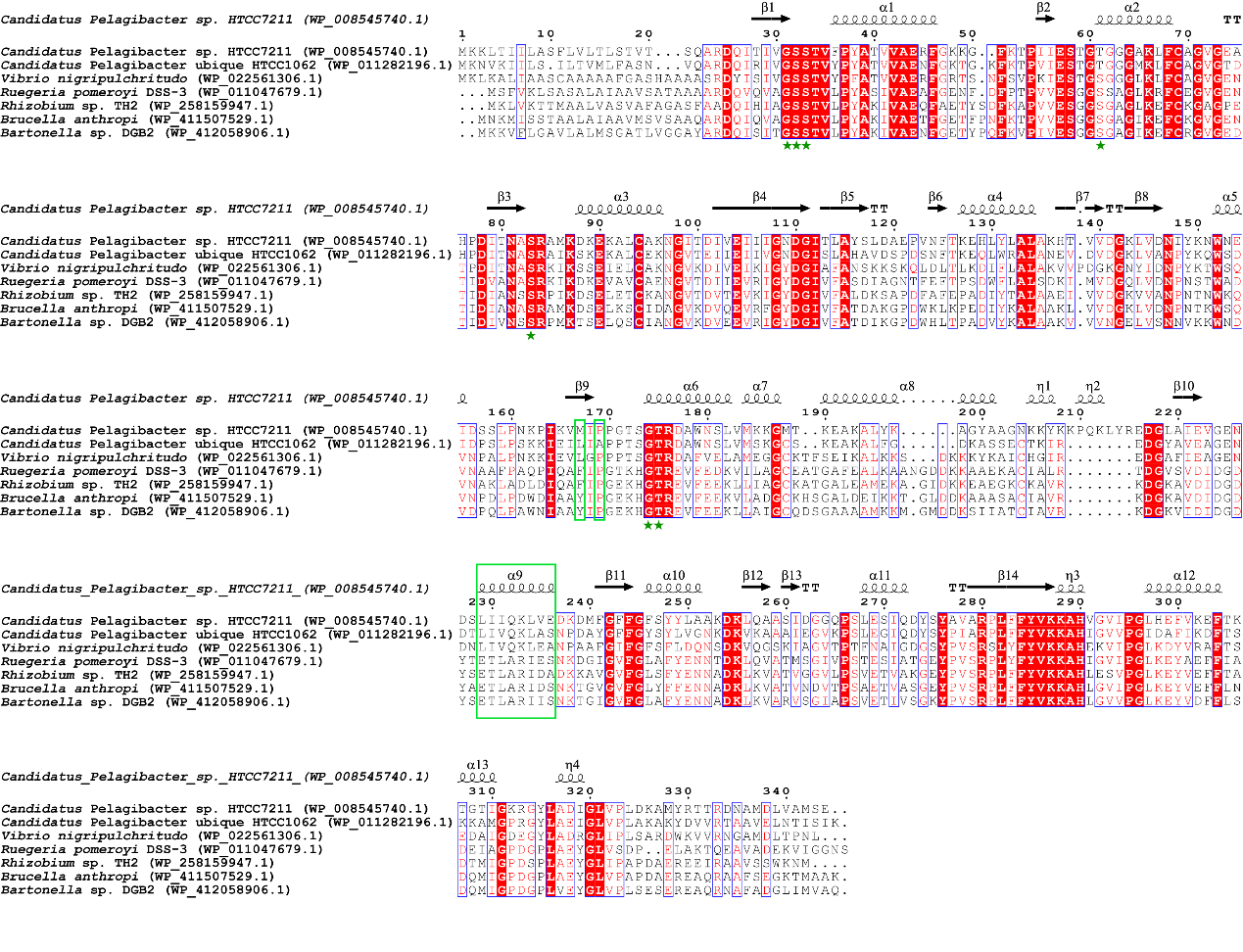


**Fig. S3 Multiple sequence alignment of PstSs classified into the same branch as *Cp*PstS.** The secondary structural elements of *Cp*PstS are shown at the top. The key residues involved in phosphate binding of *Cp*PstS are marked with green stars at the bottom of the sequence alignment. The residues in the green box are the key residues that possibly affect the dimensions of the substrate-binding cavity.

**
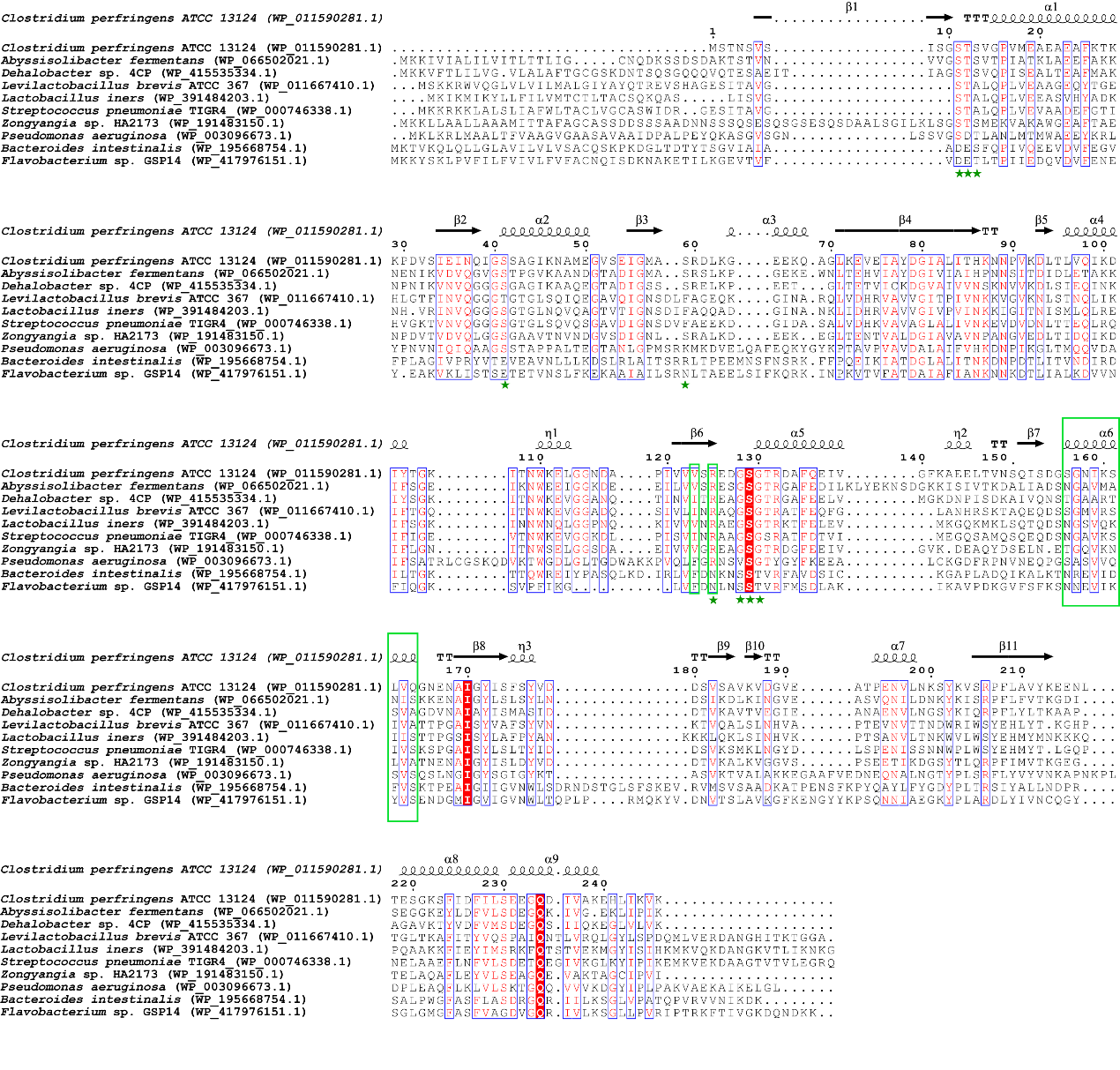
**

**Fig. S4 Multiple sequence alignment of PstSs classified into the same branch as *C. perfringens* PstS.** The secondary structural elements of *C. perfringens* PstS (PDB code: 4Q8R) are shown at the top. The key residues involved in phosphate binding of *C. perfringens* PstS are marked with green stars at the bottom of the sequence alignment. The residues in the green box are the key residues that possibly affect the dimensions of the substrate-binding cavity.


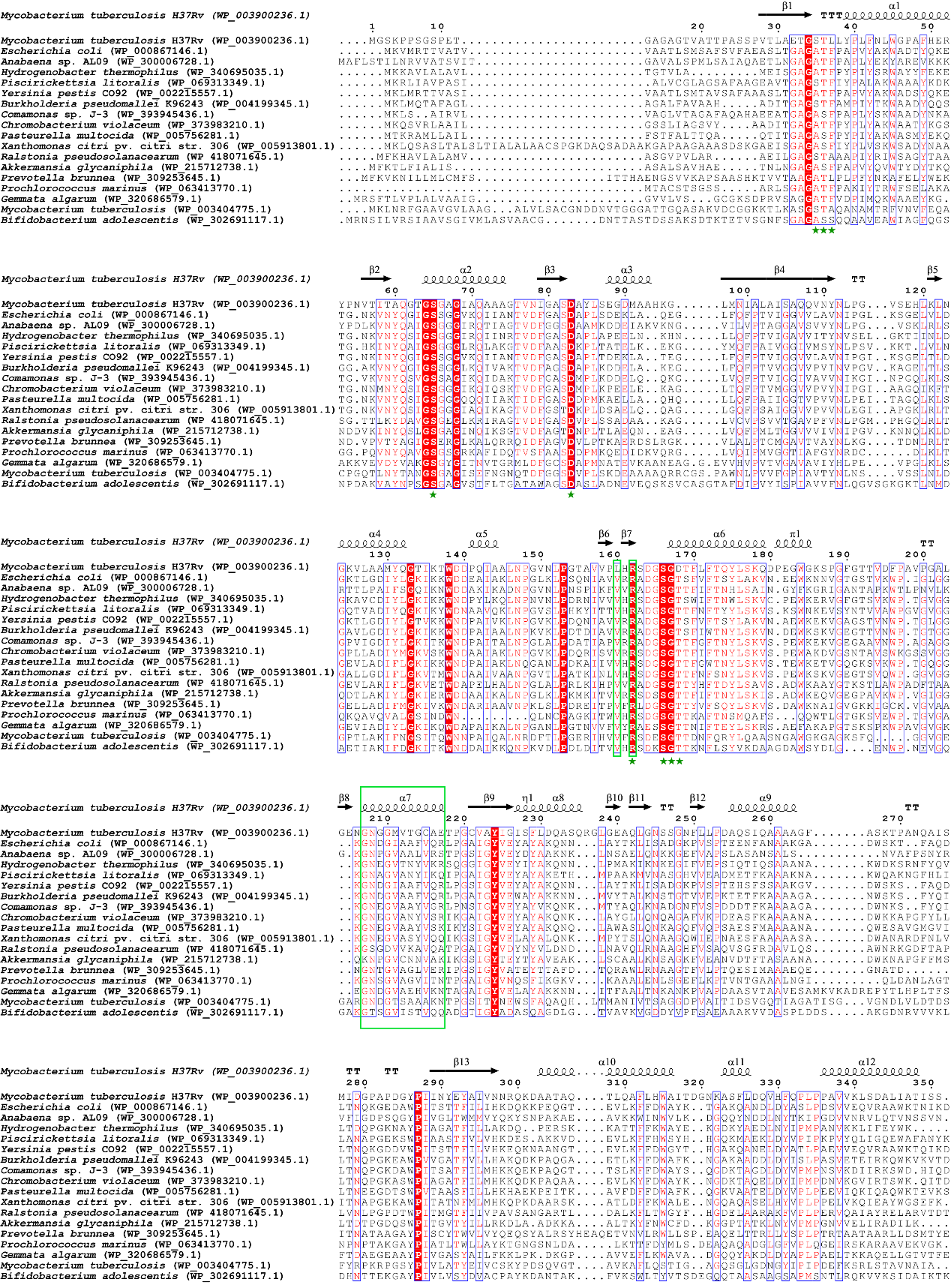


**Fig. S5 Multiple sequence alignment of PstSs classified into the same branch as *M. tuberculosis* PstS-1.** The secondary structural elements of *M. tuberculosis* PstS-1 (PDB code: 1PC3) are shown at the top. The key residues involved in phosphate binding of *M. tuberculosis* PstS are marked with green stars at the bottom of the sequence alignment. The residues in the green box are the key residues that possibly affect the dimensions of the substrate-binding cavity.


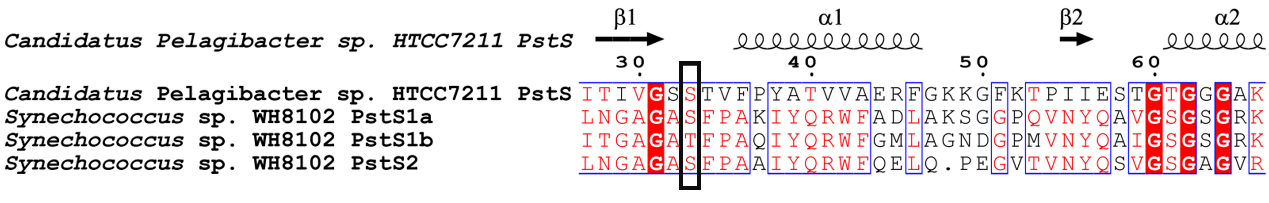


**Fig. S6 Multiple sequence alignment of *Cp*PstS with PstS1a, PstS1b, and PstS2 from Synechococcus sp. WH8102.** The secondary structural elements of *Cp*PstS are shown at the top. The residues in the black box are the key residues that possibly responsible for the higher phosphate-binding affinity of PstS in Synechococcus sp. WH8102.


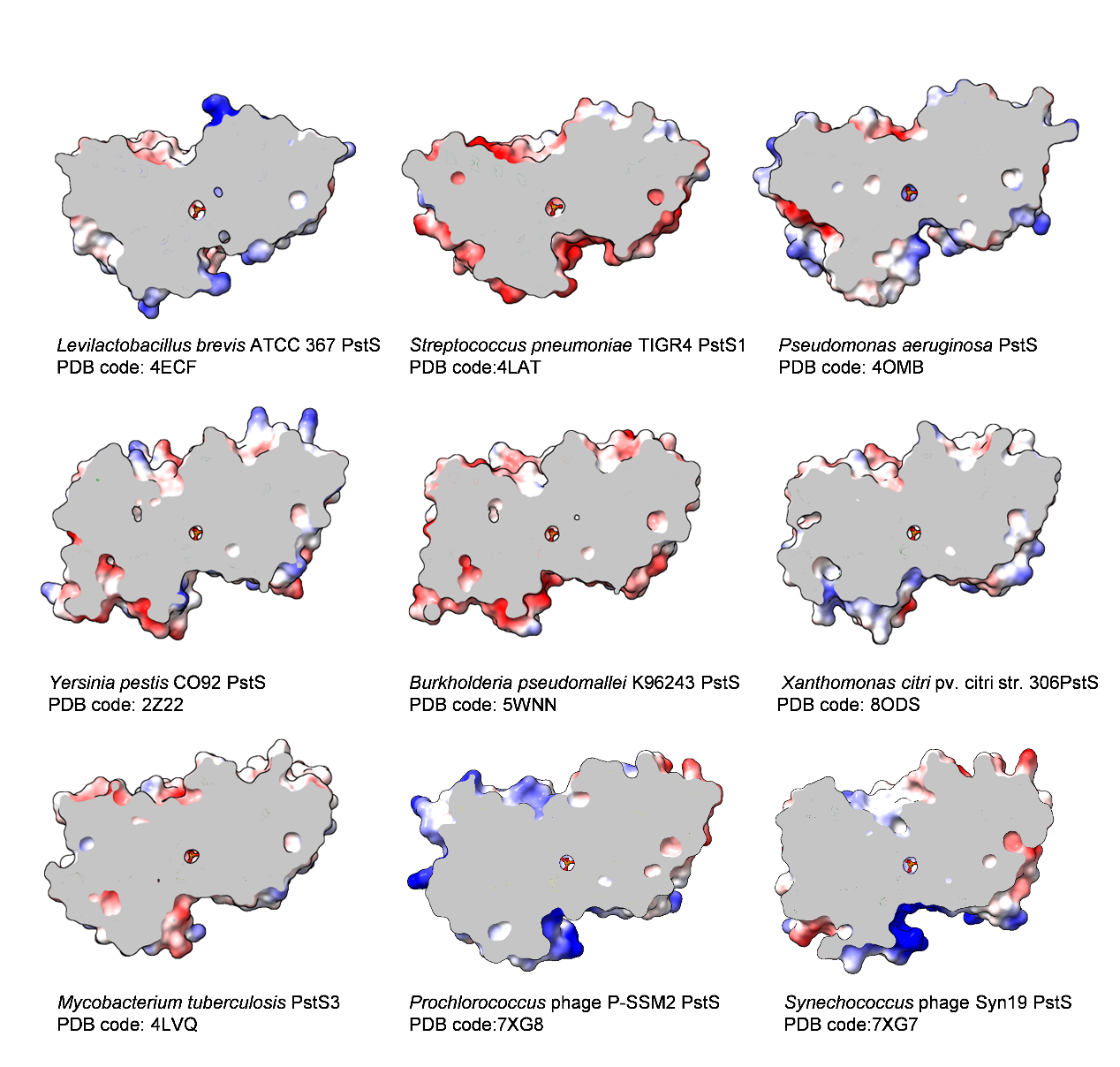


**Fig. S7 Electrostatic surface and phosphate**-**binding cavity of the nine PstS structures.** The organism and PDB code of PstSs are shown below the structure. The PstS proteins with PDB codes 7XG7 and 7XG8 are derived from phages, while the other PstS proteins are from bacteria.

**Table S1** Hydrogen bonds between phosphate and *Cp*PstS, *C. perfringens* PstS, *M. tuberculosis* PstS-1, and *E. coli* PstS.

| Pi Atom | *Cp*PstS Atom | Distance(Å) | *C. perfringens* PstS Atom | Distance  (Å) | *M. tuberculosis* PstS-1 Atom | Distance(Å) | *E. coli*  PstS Atom | Distance(Å) |
| --- | --- | --- | --- | --- | --- | --- | --- | --- |
| O1 | Ser33 N | 2.83 | Thr12 N | 2.8 | Thr36 N | 2.84 | Thr10 N | 2.76 |
| O1 | Ser33 OG | 2.76 | Thr12 OG1 | 2.6 | Thr36 OG1 | 2.61 | Thr10 OG1 | 2.64 |
| O1 | — | — | Arg125 NH2 | 2.8 | Arg162 NH2 | 2.79 | Arg135 NH2 | 2.84 |
| O2 | Thr175 N | 3.03 | Thr 131 N | 3.0 | Asp168 N | 3.08 | Thr141 N | 2.86 |
| O2 | Thr175 OG1 | 2.79 | Thr 131 OG1 | 2.6 | Asp168 OD2 | 2.54 | Thr141 OG1 | 2.68 |
| O2 | — | — | Arg 125 NH1 | 2.9 | Arg 162 NH1 | 2.83 | Arg135 NH1 | 2.83 |
| O2 | — | — | Ser129 OG | 2.7 | Ser166 OG | 2.83 | Ser139 OG | 2.66 |
| O3 | Thr61 N | 2.87 | Ser41 N | 2.8 | Ser65 N | 2.67 | Ser38 N | 2.66 |
| O3 | Thr61 OG1 | 2.73 | Ser41 OG | 2.6 | Ser65 OG | 2.74 | Ser38 OG | 2.72 |
| O3 | Gly174 N | 2.70 | Gly130 N | 2.8 | Gly 167 N | 2.73 | Gly140 N | 2.72 |
| O4 | Ser32 OG | 2.68 | Ser11 OG | 2.6 | Ser35 OG | 2.80 | — | — |
| O4 | Thr34 N | 3.09 | Ser13 N | 3.1 | Leu37 N | 3.04 | Phe11 N | 2.81 |
| O4 | Thr34 OG1 | 2.62 | Ser13 OG | 2.7 | — | — | — | — |
| O4 | Ser83 OG | 2.97 | Ser59 OG | 2.7 | Asp83 OD2 | 2.52 | Asp56 OD2 | 2.43 |

**Table S2** Bacteria and plasmids used in this study.

| Bacteria and plasmids | Genotype or property | Source |
| --- | --- | --- |
| Bacterial strains |  |  |
| *Candidatus* Pelagibacter sp. HTCC7211 | Strains for Cp*pstS* gene amplified | Da-zhi Wang Research Group, Xiamen University |
| *E. coli* BL21(DE3) | Transformed cells for gene expression | Vazyme, China |
| *E. coli* DH5α | Transformed cells for gene cloning | Vazyme, China |
| Plasmids |  |  |
| pET-22b | Plasmid for protein expression vector construction | Novagen, Germany |
| pET-22b-Cp*pstS* | Used for *Cp*PstS expression, Amp^r^, T7 promoter | This study |

**Table S3** Primers used in this study.

| Primers | Sequence (5’-3’) | Purpose |
| --- | --- | --- |
| Cp*pstS*-F | AAGAAGGAGATATACATATGAAGATCAAATTACAATCGTT | Amplification of the Cp*pstS* gene |
| Cp*pstS*-R | TGGTGGTGGTGGTGCTCGAGCTCGCTCATTGCTACAAGAT |  |

**Table S4** Phosphate-binding proteins from SAR11 bacteria.

| *Organism* | GenBank accession No. |
| --- | --- |
| *Candidatus* Pelagibacter ubique HTCC1062 | WP_011282196.1 |
| *Candidatus* Pelagibacter *sp.* IMCC9063 | WP_013694840.1 |
| *Candidatus Pelagibacter* sp. RS39 | WP_085147790.1 |
| *Candidatus* Pelagibacter sp. RS40 | WP_085147790.1 |
| *Pelagibacteraceae* bacterium GOM-A1 | OCW75579.1 |
| *Pelagibacterales* bacterium SAG-MED15 | MBD1161341.1 |
| *Pelagibacterales* bacterium SAG-MED43 | MBD1137828.1 |
| *Pelagibacterales* bacterium SAG-MED38 | MBD1139868.1 |
| *Pelagibacterales* bacterium SAG-MED28 | MBD1147158.1 |
| *Pelagibacterales* bacterium SAG-MED25 | MBD1150987.1 |
| *Pelagibacterales* bacterium SAG-MED16 | MBD1156211.1 |
| *Pelagibacterales* bacterium SAG-MED10 | MBD1165460.1 |
| *Pelagibacterales* bacterium SAG-MED07 | MBD1166368.1 |
| *Pelagibacterales* bacterium SAG-MED09 | MBD1167516.1 |
| *Pelagibacterales* bacterium SAG-MED04 | MBD1171669.1 |
| *Pelagibacterales* bacterium SAG-MED03 | MBD1173218.1 |
| *Pelagibacterales* bacterium SAG-MED05 | MBD1171952.1 |
| *Pelagibacterales* bacterium SAG-MED46 | MBD1138858.1 |
| *Pelagibacterales* bacterium SAG-MED39 | MBD1140942.1 |
| *Pelagibacterales* bacterium SAG-MED35 | MBD1142698.1 |
| *Pelagibacterales* bacterium SAG-MED14 | MBD1160145.1 |
| Alpha proteobacterium QL1 | KFX72207.1 |
| SAR11 cluster bacterium PRT-SC02 | KPU82710.1 |
